# Efficacy evaluation and exploratory analysis of influencing factors of Banxia Houpu Decoction in the treatment of refractory gastroesophageal reflux disease

**DOI:** 10.1097/MD.0000000000038045

**Published:** 2024-06-14

**Authors:** Shunzhe Song, Yunshu Zhang, Jingwen Zhang, Yongshan Jiang, Aixia Gong

**Affiliations:** a Digestive Endoscopy, First affiliated Hospital of Dalian Medical University, Liaoning, China.

**Keywords:** Banxia Houpu Decoction, gastroesophageal flap valve, proton pump inhibitors, refractory gastroesophageal reflux disease

## Abstract

Approximately 10% to 40% of patients with gastroesophageal reflux disease (GERD) exhibit poor response to proton pump inhibitors (PPIs), indicating refractory GERD (RGERD). Banxia Houpu Decoction is a traditional Chinese medicine formula used for treating GERD, particularly for atypical symptoms. This study aimed to investigate the improvement of different symptoms in RGERD patients treated with Banxia Houpu Decoction and identify relevant factors influencing its efficacy. From November 2021 to November 2022, a total of 89 RGERD patients voluntarily participated in this clinical study at our hospital. They were randomly assigned to 2 treatment groups: the Banxia Houpu Decoction group and the Western medicine group. The former received standard-dose Banxia Houpu Decoction, while the latter had a switch in PPI type with double-dose maintenance and the addition of magnesium aluminum carbonate as an acid suppressant. The improvement of different symptoms was compared between the 2 groups. Clinical data, including age, gender, gastric mucosal status, and esophagitis severity, were collected. Univariate analysis was performed to explore factors influencing the therapeutic effect of Banxia Houpu Decoction. Both treatment groups showed significant improvement in Frequency Scale for the Symptoms of GERD (FSSG) scores. The Banxia Houpu Decoction group exhibited the most significant efficacy in relieving throat burning sensation (*P = *.003) and frequent hiccups (*P* = .003). It also demonstrated improvement in swallowing difficulty (*P* = .048) and postprandial abdominal distension (*P* = .041), surpassing the Western medicine group. The Western medicine group had the most significant improvement in heartburn sensation (*P = *.008) and showed significant improvement in gastric burning sensation (*P* = .022), surpassing Banxia Houpu Decoction. Age (*P* = .025) and gastroesophageal flap valve (GEFV) grade (*P* = .014) were identified as factors influencing the efficacy of Banxia Houpu Decoction. Banxia Houpu Decoction exhibits superior efficacy compared to double-dose PPI combined with acid suppressants in relieving symptoms such as throat burning sensation, swallowing difficulty, and frequent hiccups. It shows significant efficacy in patients under 60 years of age and with GEFV grades I-II.

## 1. Introduction

Gastroesophageal reflux disease (GERD) refers to the discomforting symptoms caused by the reflux of gastric or duodenal contents into the esophagus. Typical symptoms include acid regurgitation and heartburn, but atypical symptoms such as retrosternal pressure, globus sensation, chronic cough, and chronic pharyngitis can also occur.^[1]^ The incidence of GERD varies significantly among different countries and regions worldwide, with higher rates observed in Europe, America, accounting for approximately 20% to 30% of the population. However, in recent years, the incidence of GERD in Asia has been on the rise, which may be related to improved quality of life and an increase in the proportion of obese individuals.^[2]^

Western medicine approaches to treating GERD primarily focus on suppressing gastric acid secretion. Currently, proton pump inhibitors (PPIs) are the preferred medications for GERD treatment internationally. However, studies have reported a potential association between long-term use of PPIs and osteoporosis and *Helicobacter pylori* colonization.^[3]^ Moreover, acid-suppressing drugs cannot fundamentally improve the anti-reflux barrier at the lower esophageal sphincter, and some patients exhibit poor response to PPI therapy, with approximately 30% of GERD patients classified as refractory GERD (RGERD).^[4]^ The “*2020 Chinese Expert Consensus on Gastroesophageal Reflux Disease*”^[5]^ recommends that for RGERD patients, switching to a different type of PPI while maintaining a double dose and adding an acid suppressant such as magnesium aluminum carbonate or alginate can contribute to rapid improvement of reflux and heartburn symptoms. In contrast to the single target action of Western medicine, Banxia Houpu Decoction, a traditional Chinese medicine formulation, exerts synergistic effects on different targets and is a classic formula for treating GERD, including RGERD.

Banxia Houpu Decoction is derived from Zhang Zhongjing “*Synopsis of the Golden Chamber: Miscellaneous Diseases and Pulse Manifestations*,” *and has a long history of use. It is a classic formula for treating plum pit qi, which is now recognized as globus sensation. It is believed to be an atypical symptom of GERD caused by gas reflux, resulting in increased pressure in the esophageal lumen and irritation of the upper esophageal sphincter and throat.*^[6]^ The composition of Banxia Houpu Decoction is well-designed, with Pinellia serving as the principal herb to descend counterflow and relieve nausea, Houpu (Magnolia bark) and Fuling (Poria) as ministerial herbs to regulate qi, resolve accumulation, and promote diuresis, Shengjiang (Fresh Ginger) as an assistant herb to warm the middle and relieve nausea, and Zisu (Perilla leaf) as an envoy herb to release the exterior and dispel cold. Numerous clinical studies, both domestic and international, have affirmed the therapeutic effects of Banxia Houpu Decoction in the treatment of GERD.^[7–9]^ The “*Expert Consensus on Traditional Chinese Medicine Diagnosis and Treatment of Gastroesophageal Reflux Disease*” released in 2017 also recommends Banxia Houpu Decoction as the first-choice formula for the qi stagnation and phlegm pattern of reflux disease.^[10,11]^ Banxia Houpu Decoction has been formulated into granules in Japan and is sold as an over-the-counter traditional Chinese medicine. This study aims to explore the therapeutic effects of Banxia Houpu Decoction on different symptoms in RGERD patients and the relevant factors influencing its efficacy.

## 2. Clinical data

### 2.1. General information

**Figure 1.**
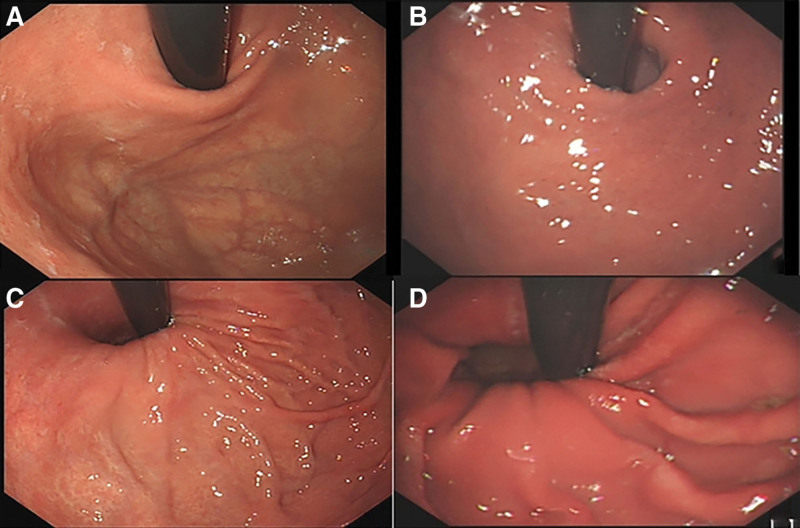
Hill classification of GEFV. (A) GEFV patient margins of mucosa fold are obvious and tightly surround the endoscope along the lesser curvature, which was considered Grade I. (B) GEFV patient mucosa fold is not obvious, the degree of compactness is not as good as that of Grade I and it is relaxed with respiratory movement, which was considered Grade II. (C) GEFV patient tissue fold can no longer wrap around the endoscopy body tightly, which was considered Grade III. (D) GEFV patient hernia sac can be seen and the dentate line of the esophagus can be seen through the hernia sac, which was considered Grade IV. GEFV = gastroesophageal flap valve.

From November 2021 to November 2022, a total of 89 patients diagnosed with RGERD at the First Affiliated Hospital of Dalian Medical University were randomly assigned to either the Banxia Houpu Decoction treatment group or the Western medicine treatment group. Patients in the Banxia Houpu Decoction group were required to visit the Traditional Chinese Medicine outpatient clinic at our hospital before treatment. Among them, 8 patients were deemed unsuitable for Banxia Houpu Decoction treatment and were excluded from this study. Ultimately, 43 patients were included in the Banxia Houpu Decoction treatment group, and 38 patients were included in the Western medicine treatment group. Baseline characteristics of both groups, including age, gender, *H pylori* infection status, gastric mucosal status, and gastroesophageal flap valve (GEFV) grade, as well as the Los Angeles classification of esophagitis, were collected. Data were recorded and compared between the 2 groups. GEFV grading was assessed according to the widely accepted Hill classification in clinical practice,^[12]^ as shown in Figure 1.

### 2.2. Diagnostic criteria

Diagnosis of GERD^[4]^: The diagnosis of GERD is based on a combination of clinical symptoms and endoscopic findings. Symptoms such as heartburn and acid regurgitation should occur at least twice a week for mild symptoms or at least once a week for severe symptoms, with or without other atypical reflux symptoms. Patients with GERD symptoms that meet these criteria should undergo thorough gastroscopy to determine whether they have erosive reflux esophagitis (RE) or non-erosive reflux disease (NERD). If there is an evident mucosal injury in the lower esophagus, it can be diagnosed as RE. If there is no evident mucosal injury in the lower esophagus, further examination using magnifying endoscopy with narrow-band imaging should be performed to observe whether there is an increase and dilation of intrapapillary capillary loops in the mucosa. If present, it can be diagnosed as NERD.^[13]^ Both RE and NERD were included in this study.

Diagnosis of RGERD^[4]^: Patients diagnosed with GERD in the outpatient setting who have undergone 4 to 8 weeks of treatment with a double dose of a particular PPI (rabeprazole 20 mg/day, lansoprazole 60 mg/day, esomeprazole 40 mg/day, or omeprazole 40 mg/day) without significant symptom relief can be diagnosed with RGERD.

### 2.3. Inclusion criteria

Age between 20 and 70 years old. Initially diagnosed with GERD in the outpatient setting and later diagnosed with RGERD after receiving 4 to 8 weeks of treatment with a double dose of PPIs without significant symptom relief. Willingness to try Banxia Houpu Decoction treatment or switch to PPI treatment with the addition of magnesium aluminum carbonate, and signing an informed consent form.

### 2.4. Exclusion criteria

Pregnant or lactating women. Patients with a history of allergies to relevant medications. Patients with severe cardiovascular, hepatic, renal, or endocrine diseases. Patients with poor compliance. Patients with a history of allergy to Chinese medicine.

## 3. Methods

### 3.1. Banxia Houpu Decoction treatment group

The Banxia Houpu Decoction treatment group received the standard dosage of Banxia Houpu Decoction, which consists of 15g of ginger (Jiang Banxia), 9g of Magnolia bark (Houpu), 12g of Poria (Fuling), 15g of fresh ginger (Shengjiang), and 6g of Perilla leaf (Zisu).^[14]^ All Chinese herbal medicines were obtained from the Chinese Medicine Pharmacy of the First Affiliated Hospital of Dalian Medical University and prepared using a professional Chinese medicine decoction machine operated by trained personnel from the Chinese Medicine Pharmacy. The decoction was prepared with 200 mL of water and divided into 2 doses to be taken orally in the morning and evening, with each dose being 100 mL. The treatment duration was 2 weeks.

### 3.2. Western medicine treatment group

According to the “*2020 Chinese Expert Consensus on Gastroesophageal Reflux Disease*” the Western medicine treatment group switched to a different PPI (rabeprazole, lansoprazole, esomeprazole, or omeprazole) while maintaining a double dose orally.^[15]^ Additionally, they received magnesium aluminum carbonate (0.5g) as an acid suppressant after each meal. The treatment duration was also 2 weeks.

### 3.3. Comparison of symptom improvement between the 2 groups

Patients in both groups completed the Frequency Scale for the Symptoms of GERD (FSSG) questionnaire^[16]^ before and after treatment. The scores for reflux syndrome (RS) and acid-related dyspepsia (ARD), as well as the scores for 12 individual symptoms, were recorded and compared between the 2 groups using t-tests to determine the treatment effects on different symptoms.

### 3.4. Identification of potential factors influencing the efficacy of Banxia Houpu Decoction

Baseline clinical information was collected for patients in both groups, including gender, age, endoscopic gastric mucosal status (atrophy, erosion, bile reflux), esophagitis grade, and GEFV grade. Univariate analysis was performed to explore potential factors that may influence the treatment efficacy.

### 3.5. Statistical methods

Data processing was performed using SPSS 19.0 statistical software. Measurement data were expressed as mean ± standard deviation, and count data were expressed as percentages (%). Data analysis included t-tests, chi-square tests, and univariate correlation analysis. A significance level of *P* < .05 was used to determine statistical significance.

## 4. Results

### 4.1. Overall treatment efficacy of the 2 groups

As shown in Table 1, out of the 89 patients, 8 patients were deemed unsuitable for Banxia Houpu Decoction treatment and were excluded from this study, resulting in a total of 81 patients included in the analysis. Among them, 43 patients were assigned to the Banxia Houpu Decoction treatment group, and 38 patients were assigned to the Western medicine treatment group. There were no statistically significant differences in baseline characteristics between the 2 groups. Both treatment groups showed significant improvement in FSSG scores. As shown in Table 2, the Banxia Houpu Decoction group showed a decrease in FSSG score from 18.90 to 12.41 (*P* < .001), with RS score decreasing from 11.14 to 7.02 (*P* = .001) and ARD score decreasing from 7.76 to 5.39 (*P* = .001). The Western medicine treatment group showed a decrease in FSSG score from 16.21 to 11.89 (*P* = .006), with RS score decreasing from 8.68 to 6.84 (*P* = .031) and ARD score decreasing from 6.81 to 5.05 (*P* = .044).

**Table 1:** Comparison of baseline characteristics between the 2 groups.

	Banxia Houpu Decoction treatment group (n = 43)	Western medicine treatment group (n = 38)	*P*
Age	55.09 ± 14.19	54.55 ± 11.68	.853
Gender (male)	19(44.19)	22(57.89)	.293
HP infection	22(51.16)	20(52.63)	.895
Gastric mucosal atrophy			.911
Non-atrophic	21(48.84)	20(52.63)	
Mild atrophy (C-1,2)	19(44.19)	15(39.47)	
Severe atrophy (C-3 and above)	3(6.98)	3(7.89)	
Gastric mucosal Erosion or protruding erosion	11(25.58)	14(36.84)	.274
Classification of reflux esophagitis			.110
M	32(74.42)	19(50.00)	
A	9(20.93)	13(34.21)	
B	2(4.65)	5(13.16)	
C	0(0.00)	1(2.63)	
D	0(0.00)	0(0.00)	
GEFV classification			.210
I	22(51.16)	11(28.95)	
II	12(27.91)	15(39.47)	
III	4(9.30)	7(18.42)	
IV	5(11.63)	5(13.16)	

GEFV = gastroesophageal flap valve, HP infection = *H pylori* infection status.

**Table 2:** Improvement in FSSG scores after treatment in the 2 groups.

FSSG related indicators	Banxia Houpu Decoction treatment group			Western medicine treatment group		
	Before medication	After medication	*P*	Before medication	After medication	*P*
Total score	18.90 ± 8.09	12.41 ± 4.60	<.001	16.21 ± 7.63	11.89 ± 5.51	.006
RS score	11.14 ± 5.77	7.02 ± 3.33	.001	8.68 ± 3.94	6.84 ± 3.33	.031
ARD score	7.76 ± 3.71	5.39 ± 2.23	.001	6.81 ± 4.42	5.05 ± 2.95	.044
FSSG Specific Projects						
Heartburn	2.14 ± 1.13	1.61 ± 0.82	.031*	1.55 ± 0.97	1.03 ± 0.68	.008**
Bloating	1.51 ± 1.03	1.09 ± 0.81	.057	1.50 ± 1.20	1.13 ± 0.81	.122
Bloating after eating	1.39 ± 1.03	0.95 ± 0.75	.041*	1.26 ± 1.13	1.13 ± 0.93	.582
Rub your upper abdomen with your hands	1.21 ± 1.21	0.84 ± 0.84	.214	0.84 ± 1.03	0.71 ± 0.80	.536
Discomfort after meals	1.88 ± 1.03	1.40 ± 0.76	.018*	1.18 ± 1.11	0.74 ± 0.69	.038*
Stomach burning	2.05 ± 1.11	1.56 ± 0.88	.061	1.39 ± 1.10	0.89 ± 0.73	.022*
Pharyngeal burning sensation	1.67 ± 1.52	0.74 ± 0.95	.003**	1.26 ± 0.89	1.11 ± 0.80	.418
Feeling full when eating	1.53 ± 0.96	1.16 ± 0.69	.077	0.89 ± 1.16	0.74 ± 0.89	.507
Swallowing choking sensation	0.98 ± 1.20	0.44 ± 0.59	.048*	1.16 ± 1.03	1.08 ± 0.97	.731
Bitter or acidic fluid backing up in the throat	1.30 ± 1.28	0.65 ± 0.78	.017*	1.45 ± 1.06	0.97 ± 0.72	.025*
Frequent hiccups	1.44 ± 1.10	0.79 ± 0.64	.003**	1.47 ± 1.08	1.31 ± 0.90	.493
Heartburn when bending over	1.74 ± 1.07	1.19 ± 0.79	.015*	1.21 ± 1.04	1.05 ± 0.87	.476

ARD = acid-related dyspepsia score, FSSG = frequency scale for the symptoms of GERD, RS = Reflux Symptom Score. (*) & (**) chi-square test.

* *P* < .05;

** *P *< .001.

### 4.2. Improvement in different symptoms as assessed by FSSG in the 2 treatment groups

As shown in Table 2, the Banxia Houpu Decoction treatment group showed significant improvement in symptoms of throat burning sensation (*P* = .003) and frequent hiccups (*P* = .003), with no significant improvement observed in the Western medicine treatment group. Additionally, significant improvements were observed in symptoms of swallowing difficulties (*P* = .048) and postprandial abdominal distension (*P* = .041) in the Banxia Houpu Decoction group, both of which were superior to the Western medicine treatment group. The Western medicine treatment group showed the most significant improvement in symptoms of heartburn (*P* = .008) and gastric burning sensation (*P* = .022), both of which were superior to the combination treatment group.

Furthermore, both treatment groups showed significant improvements in postprandial discomfort (*P* = .018, *P* = .038) and bitter or acidic fluid reflux into the throat (*P* = .017, *P* = .025). However, there were no significant improvements in symptoms of gastric bloating (*P* = .057, *P* = .122), postprandial fullness (*P* = .077, *P* = .507), and upper abdominal rubbing (*P* = .214, *P* = .536) in either group.

### 4.3. Factors influencing the efficacy of Banxia Houpu Decoction treatment

As shown in Table 3, age (*P* = .025, r = −0.342) and GEFV grade (*P* = .014, r = −0.374) were identified as factors influencing the efficacy of Banxia Houpu Decoction treatment. Both age and GEFV grade showed a negative correlation with the treatment efficacy of Banxia Houpu Decoction, indicating that younger patients with lower GEFV grades had better treatment responses to Banxia Houpu Decoction.

**Table 3:** Univariate correlation analysis of factors influencing the efficacy of Banxia Houpu Decoction treatment (improvement in FSSG scores).

Influencing factors	Correlation coefficient (Pearson or spearman)	*P*
Gender	−0.023	.882
Age	−0.342	.025
Classification of Reflux Esophagitis (LA Classification)	−0.084	.593
GEFV grade	−0.374	.014
Degree of gastric mucosal atrophy (Kimura-Takemoto classification)	−0.162	.300
HP infection	−0.173	.266
Gastric mucosal erosion or protruding erosion	−0.082	.601
Whether combined with bile reflux	−0.176	.259

Notes: GEFV = gastroesophageal flap valve, HP infection = *H pylori* infection status, LA Classification = Los Angeles classification.

## 5. Discussion

Currently, the incidence of atypical symptoms in GERD, such as swallowing sensation of foreign body, belching, and recurrent rhinitis, is increasing. Some of these symptoms are related to gas and aerosol reflux, and no significant mucosal damage can be observed under endoscopy, requiring the assistance of esophageal manometry for diagnosis. These patients often have poor response to PPI treatment and are diagnosed as RGERD.^[17]^

Studies have shown that RGERD patients have simultaneous decreases in the pressure of the upper and lower esophageal sphincters, with weak acid reflux and nonacid reflux being predominant, which is also the reason for the poor response to PPI treatment.^[18]^ The “2020 Chinese Expert Consensus on Gastroesophageal Reflux Disease” suggests that for RGERD patients, it is recommended to switch to a different type of PPI and maintain double-dose treatment.

The Banxia Houpu Decoction (Pinellia and Magnolia Bark Decoction) as a traditional herbal formula has been found to have significant efficacy in improving atypical symptoms of RGERD, such as swallowing sensation of foreign body, frequent belching, etc, which are related to gas reflux.^[7,19]^ This suggests that for patients presenting with predominant symptoms of throat obstruction or belching, Banxia Houpu Decoction could be considered for treatment. In contrast, the combination of acid suppressants and acid-neutralizing agents in the Western medicine treatment group mainly improves symptoms of heartburn and gastric burning, reducing the stimulation of gastric acid on the esophageal mucosa. The 2 approaches complement each other, targeting the 2 main symptoms of “gas reflux and acid reflux” in GERD.

Wu Xuemei et al conducted a randomized controlled trial on 85 patients with GERD-related cough and found that the efficacy of the Chinese medicine group (Banxia Houpu Decoction plus modifications) was significantly better than that of the Western medicine group (domperidone plus omeprazole).^[20]^ Tang Chen randomized 81 patients with RE into a Chinese medicine treatment group and a Western medicine control group, and found that the effective rate in the Chinese medicine group was 95.10%, significantly higher than that in the control group (82.50%).^[21]^ Japanese researchers Endo M et al found that Banxia Houpu Decoction could inhibit neutrophil and macrophage infiltration in the digestive tract smooth muscle of rats, reduce the level of NF-κB in serum, and increase the level of nerve growth factor.^[22]^ A meta-analysis of 6 studies involving 411 patients^[23]^ showed that the effective rate of Banxia Houpu Decoction in the treatment of reflux-related cough was significantly better than that of the Western medicine group (domperidone and omeprazole).

Although the exact mechanism of action of Banxia Houpu Decoction has not been fully elucidated, network pharmacology analysis has identified several major pathways and targets, including the MAPK pathway, HIF pathway, PI3K pathway, calcium signaling pathway, and neural ligand-receptor interaction.^[24]^ The active ingredients in Banxia Houpu Decoction, such as Pinellia alkaloids, can act on the 5-HT pathway to regulate smooth muscle tone,^[25]^ while magnolia phenols in houpo can act on G proteins to regulate calcium ion influx and increase sphincter tone.^[26,27]^ Perilla aldehyde in size can reduce the levels of inflammatory cytokines such as IL-1β, IL-6, and TNF-α.^[28]^ Other effective components in the formula also exert synergistic effects through different targets and pathways.

The GEFV located at the gastroesophageal junction and the greater curvature of the gastric fundus, is a valve-like muscular mucosal fold that serves as an anti-reflux barrier observed under endoscopy.^[29,30]^ As shown in Figure 1, the Hill classification is widely used to evaluate the morphology of GEFV. It has been proven^[26]^ that the morphology of GEFV is significantly related to the occurrence of GERD, with GEFV of Grade III-IV being more prone to esophageal mucosal damage. The results of this study found that GEFV of Grade I-II was more sensitive to the effects of Banxia Houpu Decoction, while GEFV of Grade III-IV indicated significant damage to the anti-reflux barrier, making it difficult to improve GERD even with the use of Banxia Houpu Decoction. However, in GERD patients with relatively normal GEFV, where the anti-reflux barrier is not significantly damaged, the symptoms are more associated with neurogenic factors and are more sensitive to the effects of Banxia Houpu Decoction.

## 6. Conclusion

As a traditional herbal formula, Banxia Houpu Decoction has shown promising efficacy in improving atypical symptoms of RGERD, such as swallowing sensation of foreign body and frequent belching, which complements the treatment with PPIs and has the potential to become a new generation of GERD treatment. However, the limitation of this study is the lack of objective indicators such as esophageal manometry data to assess esophageal motility, and the FSSG scoring questionnaire is subjective and may be influenced by individual factors.

## Author contributions

**Conceptualization:** Shunzhe Song, Aixia Gong.

**Data curation:** Yunshu Zhang, Yongshan Jiang.

**Formal analysis:** Jingwen Zhang, Yongshan Jiang.

**Methodology:** Yunshu Zhang, Jingwen Zhang.

**Resources:** Aixia Gong.

**Writing – original draft:** Shunzhe Song, Aixia Gong.

**Writing – review & editing:** Shunzhe Song, Yunshu Zhang, Jingwen Zhang, Yongshan Jiang, Aixia Gong.
